# *International Journal of Environmental Research and Public Health* Best Paper Award 2014

**DOI:** 10.3390/ijerph110101192

**Published:** 2014-01-22

**Authors:** Paul B. Tchounwou, Joyce Zhou

**Affiliations:** 1 Molecular Toxicology Research Laboratory, Jackson State University, 1400 Lynch Street, Box 18750, Jackson, MS 39217, USA; E-Mail: paul.b.tchounwou@jsums.edu; 2 MDPI AG, Postfach, CH-4005 Basel, Switzerland and MDPI Branch Office, Beijing 101121, China; E-Mail: joyce.zhou@mdpi.com

*International Journal of Environmental Research and Public Health* started to institute an annual award in 2013 to recognize outstanding papers related to environmental health sciences and public health that meet the aims, scope and high standards of this journal. We are pleased to announce the second “*International Journal of Environmental Research and Public Health* Best Paper Award” for 2014. Nominations were solicited from the Editor-in-Chief and Editorial Board members, with all papers published in 2010 eligible for consideration. The following five papers were selected for recognition awards:

## Article Award:

### 1^st^ Prize

**Authors: Mai A. Elobeid, Miguel A. Padilla, David W. Brock, Douglas M. Ruden and David B. Allison**

Article title: Endocrine Disruptors and Obesity: An Examination of Selected Persistent Organic Pollutants in the NHANES 1999–2002 Data

*Int. J. Environ. Res. Public Health* 2010, *7*(7), 2988–3005; doi:10.3390/ijerph7072988

Available online: http://www.mdpi.com/1660-4601/7/7/2988

### 2^nd^ Prize

**Authors: Timothy C. Durazzo, Dieter J. Meyerhoff and Sara Jo Nixon**

Article title: Chronic Cigarette Smoking: Implications for Neurocognition and Brain Neurobiology

*Int. J. Environ. Res. Public Health* 2010, *7*(10), 3760–3791; doi:10.3390/ijerph7103760

Available online: http://www.mdpi.com/1660-4601/7/10/3760

### 3^rd^ Prize

**Authors: Howard C. Chang, Jingwen Zhou and Montserrat Fuentes**

Article title: Impact of Climate Change on Ambient Ozone Level and Mortality in Southeastern United States

*Int. J. Environ. Res. Public Health* 2010, *7*(7), 2866–2880; doi:10.3390/ijerph7072866

Available online: http://www.mdpi.com/1660-4601/7/7/2866

## Review Award:

### 1^st^ Prize

**Authors: Laura M. Plum, Lothar Rink and Hajo Hasse**

Article title: The Essential Toxin: Impact of Zinc on Human Health

*Int. J. Environ. Res. Public Health* 2010, *7*(4), 1342–1365; doi:10.3390/ijerph7041342

Available online: http://www.mdpi.com/1660-4601/7/4/1342

### 2^nd^ Prize

**Authors: Barry Bogin and Maria Ines Varela-Silva**

Article title: Leg Length, Body Proportion, and Health: A Review with a Note on Beauty

*Int. J. Environ. Res. Public Health* 2010, *7*(3), 1047–1075; doi:10.3390/ijerph7031047

Available online: http://www.mdpi.com/1660-4601/7/3/1047

## 

The prize awarding committee merits the article “Endocrine Disruptors and Obesity: An Examination of Selected Persistent Organic Pollutants in the NHANES 1999–2002 Data” as “…[utilizing] an innovative approach to highlight the role played by human exposure to endocrine-disrupting chemicals on the etiology and prevalence of obesity…”. The article “The Essential Toxin: Impact of Zinc on Human Health” reviewed “…comprehensively of the risks and benefits of zinc on human health. Good coverage of the mechanisms by which zinc acts and public health considerations…”. The article “Leg Length, Body Proportion, and Health: A Review with a Note on Beauty” is an “…important review on human development and how it is programmed through the actions of Hox genes. Good correlation between anthropogenic measures and biology. Interesting commentary on leg length and perception of beauty…”.

These five exceptional papers are valuable contributions to the *International Journal of Environmental Research and Public Health* and the public health field. On behalf of the Prize Awarding Committee and the Editorial Board of *International Journal of Environmental Research and Public Health*, we would like to congratulate these five teams for their excellent work. In recognition of their accomplishments, Drs. David B. Allison, Timothy C. Durazzo, and Howard H. Chang will receive a prize of 600 CHF, 400 CHF, and 200 CHF, respectively, and the privilege of publishing an additional paper free of charge in open access format in the *International Journal of Environmental Research and Public Health*, after normal peer-review. Drs. Hajo Hasse and Barry Bogin will be awarded the privilege of publishing an additional research paper free of charge in open access format in the *International Journal of Environmental Research and Public Health*, after normal peer-review. 

## *Prize Awarding Committee* 

### Editor-in-Chief 

**Prof. Dr. Paul B. Tchounwou**

Molecular Toxicology Research Laboratory, Jackson State University, 1400 Lynch Street, Box 18750, Jackson, MS 39217, USA

E-Mail: paul.b.tchounwou@jsums.edu

### Editorial Board Member and Guest Editor 

**Dr. Zubair Kabir**

Department of Epidemiology & Public Health, University College Cork, Cork, Ireland

E-Mail: z.kabir@ucc.ie

### Editorial Board Member 

**Prof. Dr. William A. Toscano**

Division of Environmental Health Sciences, School of Public Health, University of Minnesota, Minneapolis, MN 55455, USA

E-Mail: tosca001@umn.edu

### Editorial Board Member 

**Prof. Dr. Assaf A. Abdelghani**

Department of Environmental Health Sciences, Tulane Universiaty School of Public Health of Tropical Medicine, New Orleans, LA 70112, USA

E-Mail: assafa@tulane.edu

### Managing Editor 

**Dr. Joyce Zhou**

MDPI Beijing Office, Suite 2011, Ruidu International Center, No. 1 Cuijingbeili, Tongzhou District, Beijing 101121, China

E-Mail: joyce.zhou@mdpi.com

